# Editorial: Women in brain health and clinical neuroscience

**DOI:** 10.3389/fnhum.2024.1388801

**Published:** 2024-03-06

**Authors:** Rosalba Morese, Elizabeth Elliott, Edda Bilek, Sara Palermo

**Affiliations:** ^1^Faculty of Biomedical Sciences, Faculty of Communication, Culture and Society, USI Università della Svizzera Italiana, Lugano, Switzerland; ^2^Centre for Clinical Brain Sciences, University of Edinburgh, Edinburgh, United Kingdom; ^3^Wellcome Centre for Human Neuroimaging, University College London, London, United Kingdom; ^4^Central Institute of Mental Health, Mannheim, Germany; ^5^Department of Psychology, University of Turin, Turin, Italy; ^6^Neuroradiology Unit, Diagnostic and Technology Department, Fondazione Istituto di Ricovero e Cura a Carattere Scientifico, Istituto Neurologico Carlo Besta, Milan, Italy

**Keywords:** gender gap, brain science, clinical neuroscience, depression, language, inter-group, sensory-processing sensitivity

In the vast realm of scientific inquiry, women have played a pivotal yet often unacknowledged role in shaping our understanding of the intricate workings of the human nervous system. The research landscape, however, remains a challenging terrain for women, with < 30% of researchers worldwide being female (https://uis.unesco.org/en/topic/women-science). Long-standing biases and gender stereotypes persist, casting a shadow over the potential of countless girls and women who could contribute significantly to science, technology, engineering, and mathematics (STEM) research.

As UNESCO underlines, science and gender equality are indispensable for achieving sustainable development. Breaking free from the chains of stereotypes and biases is imperative to pave the way for a future where talent knows no gender boundaries. It is in this context that Brain Health and Clinical Neuroscience proudly presents an editorial initiative aimed at spotlighting the remarkable contributions of female researchers in the field.

This initiative recognizes that gender equality is not just a moral imperative but a strategic necessity. The RT seeks to challenge the status quo and amplify the voices of women who have been instrumental in driving scientific advancements. The editorial is not just an acknowledgment of the achievements of these remarkable researchers but a call to action to transform traditional mindsets, promote gender equality, and inspire the next generation of women to pursue careers in STEM.

The Research Topic comprises six contributions represented by three original research articles, one brief research report, one systematic review, and one hypothesis and theory article.

The long-term potentiation (LTP) study by Rygvold et al. reveals reduced LTP-like synaptic plasticity in neuropsychiatric disorders such as major depressive disorder and schizophrenia. With a large cohort of healthy adults, the research confirms that modulations in visually evoked potentials (VEP) are associated with depressive symptoms and stress, suggesting that LTP-like plasticity may serve as a vulnerability marker for the development of depressive symptoms.

Key et al. explore the iSPOT-D clinical trial database to uncover an association between auditory event-related potential responses, clinical symptoms, and also demographic features in major depressive disorder (MDD). The findings replicate an alteration of sensory, and also an alteration of attentional processes in MDD patients, with age influencing both the topographic distribution of responses and stimuli sensitivity, suggesting potential biomarkers for patient stratification in clinical trial design.

In prior research, Lock and Keong proposed a novel taxonomic framework for interpreting white matter tract profiles using diffusion tensor imaging (DTI), validated in Classic Normal Pressure Hydrocephalus (NPH). In this study, they apply the Periodic Table algorithm to analyze Complex NPH and observe widespread reductions in subcortical deep gray matter structures, supporting the retention of Classic NPH-associated imaging features and emphasizing injury patterns over individual measures in DTI interpretation.

Farshchi et al. address the limited understanding of the neurophysiological processing of negation in natural language, particularly in the context of spoken language. While previous research based on written stimuli suggested processing costs related to negation, the study developed an auditory paradigm replicating these costs but revealing distinct patterns of ERP effects. The findings suggest that processing negation in spoken language may be less effortful, possibly due to the natural flow of speech reducing variability in neural responses, as opposed to the written word-by-word paradigm.

Saarinen et al. investigate whether there is a neural inter-group bias between minority and majority members based on ethnicity and explored differential neural processing of various ethnic groups. The results indicate that ethnic minority members did not exhibit the bias, while ethnic majority members displayed biased responses toward minority members in brain regions associated with several neural processes such as facial elaboration, and attention.

Morellini et al. propose that highly sensitive people may be particularly susceptible to social exclusion conditions beyond the physical pain, offering insights for developing interventions to support their wellbeing. While not pathological, sensory-processing sensitivity (SPS) may increase vulnerability to risk conditions during vulnerable life periods ad childhood and adolescence.

In celebrating the achievements of *Women in brain health and clinical neuroscience*, we not only honor their individual contributions but also challenge the prevailing norms that hinder the full realization of their potential. Our continually evolving neurocognitive model, grounded in advancements concerning social cognition (Morese and Palermo, 2020, 2022), metacognitive-executive functions and their intricate interactions with neurodegenerative diseases (Morese et al., 2018; Palermo et al., 2018), acquired brain injuries and pathologies (Palermo et al., 2014, 2019), represents a step toward creating a future where talent is recognized and celebrated regardless of gender. This approach fosters an environment where the scientific community thrives on the diverse perspectives and experiences that women bring to the table, emphasizing the importance of inclusivity and equity in advancing research and innovation.

## Author contributions

RM: Writing – review & editing. EE: Writing – review & editing. EB: Writing – review & editing. SP: Conceptualization, Supervision, Writing – original draft.

## References

[B1] Morese R. Palermo S. (2020). Altruistic punishment and impulsivity in Parkinson's disease: a social neuroscience perspective. Front. Behav. Neurosci. 14:102. 10.3389/fnbeh.2020.00102 32792921 PMC7385270

[B2] Morese R. Palermo S. (2022). Feelings of loneliness and isolation: social brain and social cognition in the elderly and Alzheimer's disease. Front. Aging Neurosci. 14:896218. 10.3389/fnagi.2022.896218 35936772 PMC9353216

[B3] Morese R. Stanziano M. Palermo S. (2018). Commentary: metacognition and perspective-taking in Alzheimer's disease: a mini-review. Front. Psychol. 9:2010. 10.3389/fpsyg.2018.02010 30405494 PMC6204392

[B4] Palermo S. Andò A. Salatino A. Sirgiovanni S. De Faveri L. Carassa A.. (2019). Selective emotional dysregulation in splenium agenesis. A case report of a patient with normal cognitive profile. Front. Psychol. 10:631. 10.3389/fpsyg.2019.00631 30967819 PMC6438861

[B5] Palermo S. Leotta D. Bongioanni M. R. Amanzio M. (2014). Unawareness of deficits in ischemic injury: role of the cingulate cortex. Neurocase. 5, 540–555. 10.1080/13554794.2013.826686 23962086

[B6] Palermo S. Stanziano M. Morese R. (2018). Commentary: anterior cingulate cortex and response conflict: effects of frequency, inhibition and errors. Front. Behav. Neurosci. 12:171. 10.3389/fnbeh.2018.00171 30138490 PMC6092509

